# Carbonic Anhydrase Activators. Part 19^1^ Spectroscopic and Kinetic Investigations for the Interaction of Isozymes I and II With Primary Amines

**DOI:** 10.1155/MBD.1997.221

**Published:** 1997

**Authors:** Fabrizio Briganti, Andrea Scozzafava, Claudiu T. Supuran

**Affiliations:** Università degli Studi Laboratorio di Chimica Inorganica e Bioinorganica Via Gino Capponi 7 Firenze I-50121 Italy

## Abstract

The interactions of Zn(II)- and Co(II)-substituted carbonic anhydrase (CA) isozymes I and II with amine type activators such as histamine, serotonin, phenetylamine dopamine and benzylhydrazine have been investigated kinetically, and spectroscopically. All of such activators are of the non-competitive type towards CO_2_ hydration and 4-nitrophenylacetate hydrolysis for both human isozymes (HCA I and HCA II). The electronic spectra of the adducts of Co(II)CA with amine activators are similar to the spectrum of the previously reported Co(II)CAII-phenol adduct, the only known competitive inhibitor towards CO_2_ hydration, where the phenol molecule binds into the hydrophobic pocket of the active site. This is a direct spectroscopic evidence that the activator molecules bind within the active site, but not directly to the metal ion. Recent X-ray crystallographic data for the adduct of HCA II with histamine show that the activator molecule is bound at the entrance of the active site cavity, near to residues His 64, Asn 62 and Gln 92, where actively aids in shuttling protons between the active site and the environment. Similar arrangements probably occur for the other activators reported in the present paper.


### CARBONIC ANHYDRASE ACTIVATORS. PART 19[1] SPECTROSCOPIC AND KINETIC INVESTIGATIONS FOR THE INTERACTION OF ISOZYMES I AND II WITH PRIMARY AMINES

Fabrizio Briganti, Andrea Scozzafava and Claudiu T. Supuran*

Università degli Studi, Laboratorio di Chimica Inorganica e Bioinorganica,
Via Gino Capponi 7, I-50121 Firenze, Italy.

**Abstract:** The interactions of Zn(II)- and Co(II)-substituted carbonic anhydrase (CA) isozymes I and II with amine type activators such as histamine, serotonin, phenetylamine dopamine and benzylhydrazine have been investigated kinetically, and spectroscopically. All of such activators are of the non-competitive type towards $CO_2$ hydration and 4-nitrophenylacetate hydrolysis for both human isozymes (HCA I and HCA II). The electronic spectra of the adducts of Co(II)CA with amine activators are similar to the spectrum of the previously reported Co(II)CAII-phenol adduct, the only known competitive inhibitor towards $CO_2$ hydration, where the phenol molecule binds into the hydrophobic pocket of the active site. This is a direct spectroscopic evidence that the activator molecules bind within the active site, but not directly to the metal ion. Recent X-ray crystallographic data for the adduct of HCA II with histamine show that the activator molecule is bound at the entrance of the active site cavity, near to residues His 64, Asn 62 and Gln 92, where actively aids in shuttling protons between the active site and the environment. Similar arrangements probably occur for the other activators reported in the present paper.

**Introduction**

Carbonic anhydrase (CA, EC 4.2.1.1), a zinc enzyme widely spread in the bacterial, vegetal and animal kingdoms,[2,3] catalyzes one of the simplest physiological reactions, the reversible interconversion between $CO_2$ and the bicarbonate ion.[3-6] Although at least eight distinct isozymes (CA I-VIII) are presently known in higher vertebrates, together with two CA-like proteins (CA IX and CA X),[7] the physiological function for many of them is still unknown.[3]

Activators of isozymes HCA I and II possessing the general formula **1** (ArCH($R^3$)CH($R^2$)NH$R^1$ ($R^3$ = H, OH, COOH; $R^1$ = $R^2$ =H, Me) have recently been described,[8-10] and kinetic measurements were done both for the hydrase[11] as well as esterase activity, proving a non-competitive type of interaction.[12] It is generally assumed that activators facilitate the rate-determining step in catalysis, which is a proton transfer reaction, delineated below by equations *2* and *3*.[8-12]

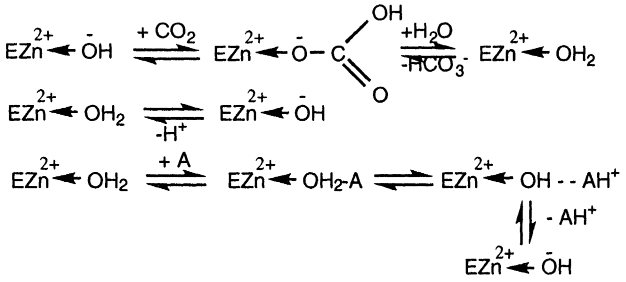

*(1)* *(2)* *(3)*

enzyme - activator (E-A) complexes

The above mechanism proposed earlier by one of us,[8] has recently been confirmed after the report of the X-ray crystallographic structure of the adduct of histamine, an effectiveCA activator, with HCA II[13] as well as that of the ternary complex of HCA II with phenylalanine and azide.[1] As seen from Fig. 1, the activator molecule is bound at the entrance of the active site cavity in a region rich in hydrophilic amino acid residues, having a suitable orientation for shuttling protons between the active site and the environment.

Taking into account the fact that biologically relevant molecules possessing the general formula **1**, others than histamine **2**, such as serotonin **3**, dopamine **4**, or phenethylamine **5** (the simplest compound possessing formula **1**) have been previously shown to activate different CA isozymes,[8-12] it appeared of interest to undergo a detailed study regarding their interaction with native and metallo-substituted CAs, in

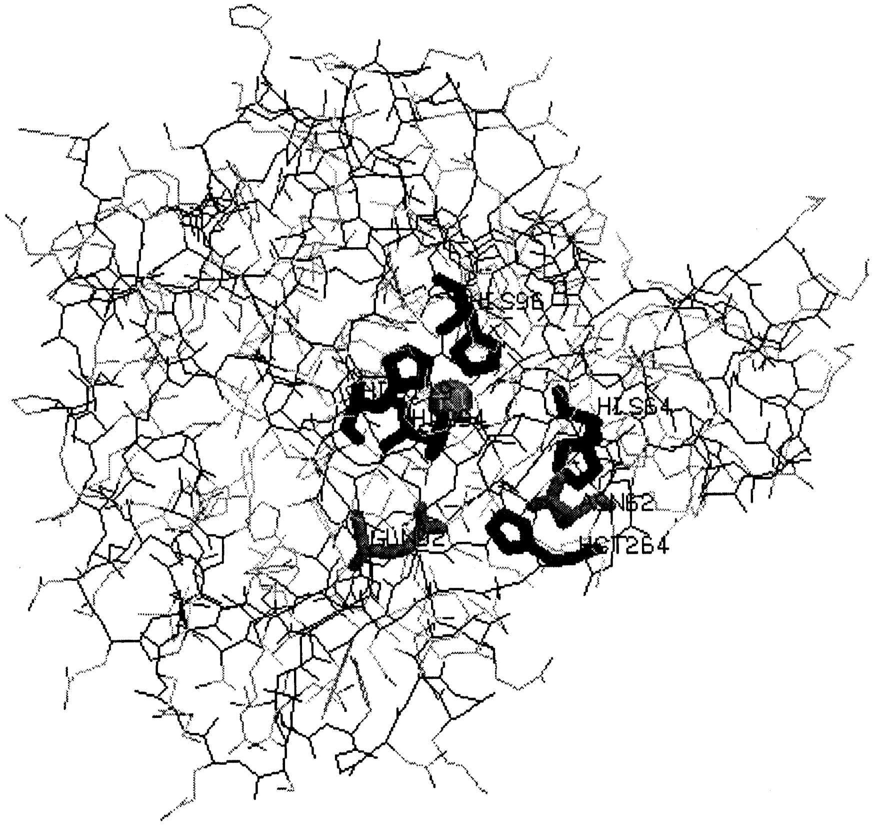

Fig. 1: HCA II - histamine adduct. The Zn(II) ion (central sphere) and its three histidine ligands (His 94, His 96 and His 119), as well as active site residues involved in histamine binding (named HST 264) such as His 64, Asn 62 and Gln 92, are evidenced. The figure was generated by using the program RasWin from the X-ray crystallographic coordinates of Briganti et al (Brookhaven Protein Database accession No. 4TST).[13]

order to verify whether their mechanism of action is similar to the one described above for histamine binding.[13] In this paper we report kinetic and spectroscopic studies regarding the interaction of four such activators (compounds **3-6**) with human isozymes I and II.

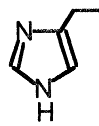

**2**

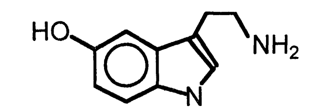

**3**

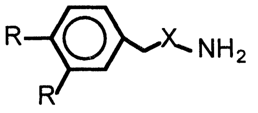

**4**: R = OH; X = $CH_2$
**5**: R = H; X = $CH_2$
**6**: R = H; X = NH

**Materials and Methods**

Human CA I and CA II cDNAs were expressed in $lon^-$ *Escherichia coli* strain BL21 (DE3) from the plasmids pACA/HCA I and pACA/HCA II[14] (the two plasmids were a gift from Prof. Sven Lindskog, Umea University, Sweden). Cell growth conditions were those described by Lindskog's group,[15] and enzymes were purified by affinity chromatography according to the method of Khalifah et al.[16] Enzyme concentrations were determined spectrophotometrically at 280 nm, utilizing a molar absorptivity of 49 $mM^{-1}.cm^{-1}$ for CA I and 54 $mM^{-1}.cm^{-1}$ for CA II, respectively, based on $M_r$ = 28.85 kDa for CA I, and 29.3 kDa for CA II, respectively.[17,18] Apoenzymes were prepared by dialysing the zinc enzymes against 50 mM pyridine-2,6-dicarboxylic acid (Sigma) in 0.2 M phosphate buffer at 4°C for 2 hours.[19] The chelating agent was removed by dialysis against 20 mM Tris-$H_2SO_4$ (pH 7.5) and then 1.1 equivalents of $CoSO_4$ were added to the apoenzymes in order to obtain Co(II)CAs. Activators (compounds **2-6**), 4-nitrophenyl acetate and solvents were from Sigma or Acros and were used without further purification.

Electronic spectra were recorded with a Cary 3 spectrophotometer interfaced with an IBM PC. Initial rates of 4-nitrophenyl acetate hydrolysis were monitored spectrophotometrically, at 400 nm and 25°C, with a Cary 3 apparatus interfaced with an IBM compatible PC. Solutions of substrate were prepared in anhydrous acetonitrile; the substrate concentrations varied between $10^{-2}$ and $10^{-5}$ M. A molar absorption coefficient $\varepsilon$ = 18400 $M^{-1}.cm^{-1}$ was used for the 4-nitrophenolate formed by hydrolysis, in the conditions of the experiments (pH 7.80), as reported by Pocker and Stone.[20] Non-enzymatic hydrolysis rates were always subtracted from the observed rates. Stock solutions of activators (10 mM) were prepared in DMSO and dilutions up to 0.01 µM were done with distilled deionized water. Duplicate experiments were done for each activator, and the values reported throughout the paper are the averages of such results. Hydratase activity in the absence and in the presence of activators has been measured by Maren's micromethod[21] at 0°C. Water saturated with 100% $CO_2$ (at 0°C) has been used as substrate. Stock solutions of activators (1 mM) were prepared in distilled-deionized water with 10-20% (v/v) DMSO for compounds possessing poor water solubility (DMSO is not inhibitory/activatory at the concentrations used in these experiments) and dilutions up to 0.01 µM were done thereafterwith distilled-deionized water. Activator and enzyme solutions were preincubated together for 10 min. at room temperature prior to assay, in order to allow for the formation of the E-A complex. In a special $CO_2$ bubbler cell 0.3 mL of distilled water was added, followed by 0.4 mL of phenol red indicator solution (1%) and (0.1 mL of inhibitor + 0.1 mL of CA solution, preincubated as mentioned above). The CA concentrations were 1.5 nM for CA II, 14 nM for CA I. The hydration reaction was initiated by addition of 0.1 mL of barbital buffer (pH 7.5), and the time to obtain a color change has been recorded with a stopwatch. Enzyme specific activity in the presence and in the absence of activators was determined as described by Maren.[21] The standard error of these measurements is around 5-10 %.[21]

**Results and Discussion**

Activation of the hydrase as well as esterase activity of HCA I and II with derivatives **2-6** is shown in table I, at concentrations of 10 µM of activator.

Table I. HCA I and II activation with compounds **2-6** at concentrations of 10 µM, for 4-nitrophenyl acetate hydrolysis (4-NPA) and $CO_2$ hydration reactions.

| Activator | % CA activity [a] | | | |
|---|---|---|---|---|
| | 4-NPA hydrolysis[b] | | $CO_2$ hydration[c] | |
| | HCA I | HCA II | HCA I | HCA II |
| **2** | 161 | 119 | 176 | 150 |
| **3** | 118 | 108 | 143 | 112 |
| **4** | 123 | 110 | 159 | 138 |
| **5** | 112 | 106 | 115 | 109 |
| **6** | 114 | 108 | 117 | 108 |

[a] CA activity in the absence of activator is taken as 100%; [b] At 25 °C; [c] At 0 °C.

As seen from data of Table I, the most potent activator in this series is histamine **2**, followed by dopamine **4** and serotonin **3**, whereas phenethylamine **5** and benzylhydrazine **6** behave as weaker activators against both isozymes. It should be noted that the activatory effect is better emphasized when working at 0°C and with $CO_2$ as substrate, than when working with 4-nitrophenyl acetate. This is probably due to small differences in the catalytic mechansims involving these two substrates. In both cases the rate determining step in catalysis is a proton transfer reaction from the zinc-bound water to the reaction medium, but presumably when the bulkier ester is bound within the active site (together with the activator molecule), the proton transfer process might be somehow less favoured as compared to the case in which $CO_2$ is acting as substrate. Isozyme I on the other hand is more susceptible to activation as compared to isozyme II, probably because it contains a smaller proportion of histidine residues as compared to HCA II, in which a cluster of such amino acids has been recently evidenced at the entrance of the active site, and which might explain its very high catalyticefficiency.[13]

In fact we have recently evidenced the fact that isozymes HCA I, HCA II and BCA IV have a very diverse behaviour towards different classes of activators.[22] It is to note that compound **5** is the first example of activator with the general formula **1**, in which a carbon atom has been substituted with one of its isosters, i.e., an NH moiety. The obtained activator is weak indeed, similarly with phenethylamine, which also activates around 110% at $10^{-5}$ M.[9]

We reported recently [23,24] that activators of the type investigated here are non-competitive with the substrate $CO_2$, for BCA and HCA I and II. As for the corresponding hydrase activity, also in the case of 4-nitrophenyl acetate hydrolysis, it was recently proved that activators of the amine type bind non-competitively to both isozymes HCA I and HCA II, in agreement with the scheme proposed for explaining their mechanism of action (equation *3*).[24,25] In order to verify the kinetic measurements mentioned above, the electronic spectra of the adducts of these activators with Co(II)-CA were measured. It is known that the Co(II) ion in Co(II)-CA is a good spectroscopic probe for the interaction of the enzyme with its substrates and inhibitors.[26-28] Electronic spectra of Co(II)-HCA II and its adducts with some activators investigated here are shown in Figs. 2 and 3.

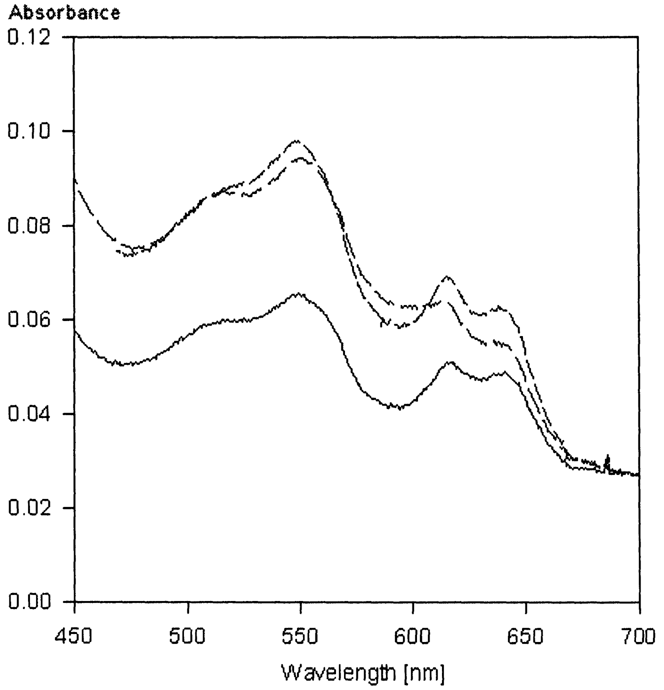

Fig. 2: Electronic spectra of Co(II)-HCA II (continuous line) and its adducts with serotonin **3** and benzylhydrazine **6** (uppermost spectrum). Conditions were: enzyme concentration 0.4 mM in all experiments; activator concentrations were : [serotonin] = 4.1 mM; [benzylhydrazine]= 1.9 mM. pH was maintained constant at 7.20.

From the data of Figs 2 and 3, it can be seen that slight differences appear between the spectra of the enzyme-activator adducts, as compared to the spectrum of pure Co(II)HCA II at the same pH. These spectra on the other hand are not similar with those of any known anionic or sulfonamide CA inhibitor.[26-28] The only spectrum to which they are similar is that of the adduct of Co(II)-CA II with phenol, the only reported competitive inhibitor with $CO_2$ as substrate of this isozyme.[29] This inhibitor has been shown to bind in the hydrophobic pocket of the enzyme, without displacing the metal-bound solvent molecule. This binding has been confirmed recently after the X-ray structure of the adduct has been reported by Nair et al.[30] Phenol does not coordinate to zinc, but binds the zinc-bound solvent through a 2.6 Å hydrogen bond, and a second, poorly oriented hydrogen bond has also been detected between the phenolic hydroxyl and the NH of Thr-199 (of 3.2 Å).

The close resemblance between the electronic spectra of our adducts with CA activators, and that of phenol, strongly suggests that the activators investigated here bind to the enzyme in a similar manner to phenol,[30] and to histamine[13] i.e., without displacing the zinc-bound solvent molecule. In this way, they are able to participate in efficient proton-shuttling processes between the active site and the medium.

An exception to the above-mentioned scheme is dopamine **4**. Initially, the electronic spectrum of the adduct of this activator with CoCA II is very similar to those of adducts with histamine, serotonin, benzylhydrazine or phenol (Fig. 3). But after 5 minutes, changes are already obvious in this spectrum. Such changes take place for at least 45 minutes. After that time the spectrum has completely changed, being similar to that of adducts with CA inhibitors.[26-28] (Fig 3).

In order to explain this phenomenon, we have redetermined activity after incubating HCA II with dopamine for different periods (up to 24 hours), considering that initially an enzyme-activator complex forms between dopamine and CA II, in which the activator molecule binds to the metal-bound solvent, and shuttles protons, by the general scheme explained above, but lately, another type of interaction might be favored between the enzyme and dopamine, by which this compound directly binds to the metal ion, by means of the two phenolic OH moieties. Such a binding has already been proposed by us for pyridinium derivatives of dopamine, of type **7**.[31] Such compounds were prepared in order to see whether the presence of $NH_2$ groups is necessary in the molecule of a compound in order to act as CA activator.[11,12] The modified derivatives **7** not only did not activate CA II, but they were inhibitors, and the mode of binding shown bellow was proposed.

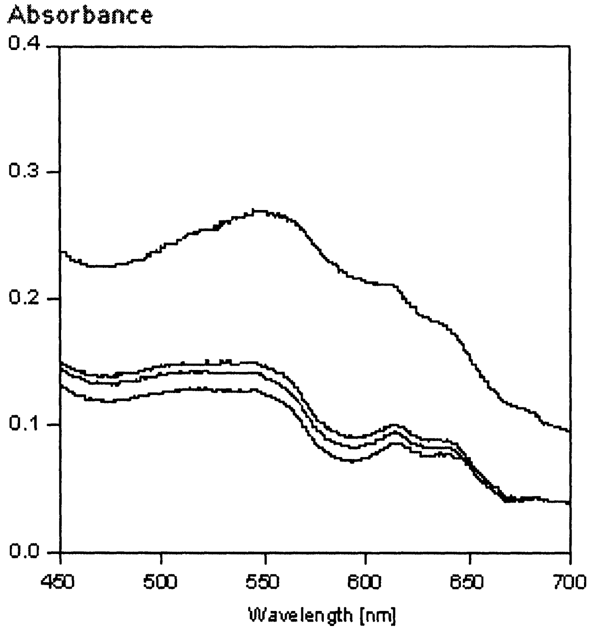

Fig. 3: Electronic spectrum of dopamine **4** (2.4 mM) and Co(II)-HCA II (0.4 mM), in 50 mM Hepes buffer, pH 7.60. The first spectrum in the lower part is registered at the initial time, the next one after 5 min, then after 10 min, and the upper spectrum was obtained after 45 min.

As seen from data of Figure 4, the longer the incubation time of enzyme and dopamine, the stronger is the activation observed. Thus, the mode of binding by means of the hydroxyl moieties may be excluded for the zinc enzyme, but probably occurs in the case of the Co(II) derivative, taking into account the large modifications observed in the electronic spectrum of this adduct. This is the first example in which a compound binds differentlyto the Zn(II)- and Co(II)-containing CAs, respectively, a fact that might also explain discrepancies between spectroscopic [26-28] and crystallographic[32] data of adducts of these enzymes with anion inhibitors, which has been explained by us for the case of cyanate binding. [5]

But activators of these widely spread enzymes, presumably possess important physiological functions too. Of the compounds investigated by us here, at least three, histamine, serotonin and dopamine, are important autacoids, present in concentrations high enough to elicit CA activation in many tissues.

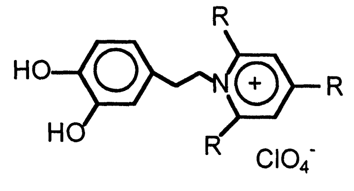

**7**: R = alkyl, aryl

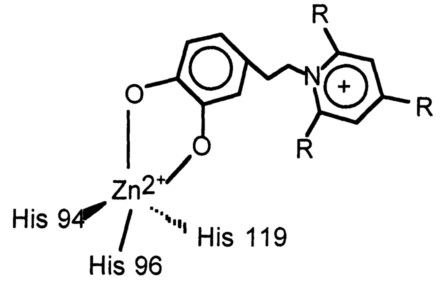

More than that, CA activation has also been reported with amino acids and oligopeptides, some of which are important.[23] Although hypothesis have been made[33] regarding the role of such activators in intracellular signal-transducing systems, more detailed studies are needed in order to understand the role played by CA activators *in vivo*, in physiologic as well as physiopathologic conditions, now that the mechanism of action at molecular level of CA activators has been probed.

**Acknowledgments.** Financial support for CTS from the T.H. Maren Scientific Foundation is greatly acknowledged.

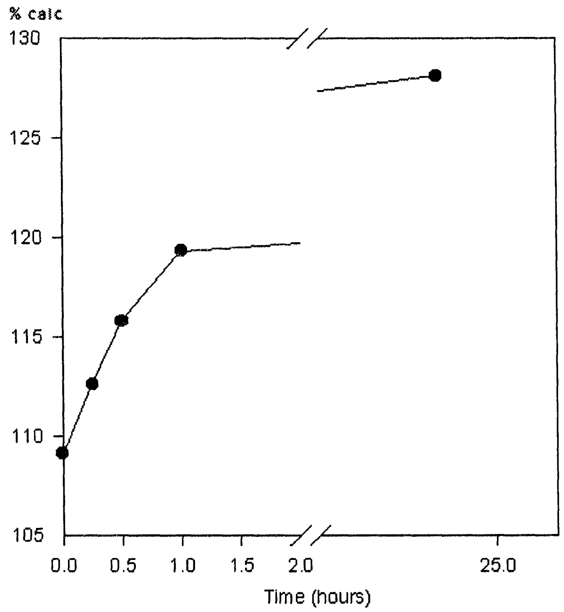

Fig. 4: Effect of dopamine incubation with HCA II upon activation of 4-nitrophenyl acetate hydrolysis. Conditions were: [HCA II] = 0.4 μM; the dopamine concentration was 0.01 mM; substrate concentration: 0.1 mM; 50 mM Tris buffer, pH 7.60, ionic strength 0.1 ($K_2SO_4$), at 25°C.

**References**

1. Part 18 of this series: F. Briganti, V. Iaconi, S. Mangani, P. Orioli, A. Scozzafava, G. Vernaglione, C.T. Supuran, *Inorg. Chim. Acta*, in press.
2. T.H.Maren, *Physiol. Rev.*, **1967**, *47*, 595-782.
3. C.T.Supuran, "Carbonic anhydrase inhibitors", in *Carbonic Anhydrase and Modulation of Physiologic and Pathologic Processes in the Organism*, I.Puscas Ed., Helicon, Timisoara **1994**, pp. 29-111.
4. S.Lindskog and A.Liljas, *Curr. Opin. Struct. Biol.*, **1993**, *3*, 915-920.
5. C.T.Supuran, C.W.Conroy and T.H.Maren, *Proteins*, **1997**, *27*, 272-278.
6. D.W. Christianson, C.A. Fierke, *Acc. Chem. Res.*, **1996**, *29*, 331-339.
7. D. Hewett-Emmett, R.E. Tashian, *Mol. Phylogenet. Evol.*, **1996**, *5*, 50-77.
8. I. Puscas, C.T. Supuran, G. Manole, *Rev. Roum. Chim.*, **1990**, *35*, 683-689.
9. B.W. Clare, C.T. Supuran, *J. Pharm. Sci.*, **1994**, *83*, 768-773.
10. C.T. Supuran, A.T.Balaban, P.Cabildo, R.M.Claramunt, J.L.Lavandera, J.Elguero, *Biol.Pharm.Bull.*, **1993**, *16*, 1236-1239.
11. C.T.Supuran, M.Barboiu, C.Luca, E.Pop,M.E.Brewster, A.Dinculescu, , *Eur. J.Med.Chem.*, **1996**, 31, 597-606.
12. C.T.Supuran, R.M.Claramunt, J.L.Lavandera, J.Elguero, *Biol. Pharm. Bull.*, **1996**, *19*, 1417-1423.
13. F. Briganti, S. Mangani, P. Orioli, A. Scozzafava, G. Vernaglione, C.T. Supuran, *Biochemistry*, **1997**, in press.
14. C.Forsman, G.Behravan, A.Osterman and B.H.Jonsson, *Acta Chem. Scand.*, **1988**, *B42*, 314-318.
15. G.Behravan, P.Jonasson, B.H.Jonsson and S.Lindskog, *Eur.J.Biochem.*, **1991**, *198*, 589-592.
16. R.G.Khalifah, D.J.Strader, S.H.Bryant and S.M.Gibson, *Biochemistry*, **1977**, *16*, 2241-2247.
17. P.O.Nyman and S.Lindskog, *Biochim.Biophys.Acta*, **1964**, *85*, 141-151.
18. L.E.Henderson, D.Henriksson and P.O.Nyman, *J.Biol.Chem.*, **1976**, *251*, 5457-5463.
19. J.B.Hunt, M.J.Rhee and C.B.Storm, *Anal.Biochem.*, **1977**, *79*, 614-617.
20. Y.Pocker and J.T.Stone, *Biochemistry*, **1967**, *6*, 668-679.
21. T.H. Maren, *J.Pharmacol. Exp. Ther.*, **1960**, *130*, 26-30.
22. M.A. Ilies, M.D. Banciu, M. Ilies, F. Chiraleu, F. Briganti, A. Scozzafava, C.T. Supuran, *Eur. J.Med.Chem*, **1997**, *32*, in press.
23. Puscas, I., and Supuran, C.T. "Carbonic anhydrase activators", in *Carbonic Anhydrase and Modulation of Physiologic and Pathologic Processes in the Organism*, I. Puscas, Ed., Helicon, Timisoara **1994**, pp. 112-146.
24. C.T. Supuran, A.T. Balaban, *Rev.Roum.Chim.*, **1994**, *39*, 107-113.
25. M. Coltau, I. Puscas, C.T. Supuran, *Rev.Roum.Chim.*, **1994**, *39*, 457-462.
26. I. Bertini, G. Canti, C. Luchinat, A. Scozzafava, *J.Am.Chem.Soc.*, **1978**, *100*, 4873-4877.
27. I. Bertini, C. Luchinat, *Acc.Chem.Res.*, **1983**, *16*, 272-277.

28. I. Bertini, C. Luchinat, A. Scozzafava, *Struct.Bonding*, **1982**, *48*, 45-92.
29. I. Simonsson, B.H. Jonsson, S. Lindskog, *Biochem.Biophys.Res.Commun.*, **1982**, *108*, 1406-1412.
30. S.K. Nair, P.A. Ludwig, D.W. Christianson, *J.Am.Chem.Soc.*, **1994**, *116*, 3659-3660.
31. C.T. Supuran, I. Baciu, A.T. Balaban, *Rev.Roum.Chim.*, **1993**, *38*, 725-732.
32. M. Lindahl, L.A. Svensson, A. Liljas, *Proteins, Struct., Fuct., Genet.*, **1993**, *15*, 177-182.
33. Puscas, I., and Supuran, C.T. " Carbonic anhydrase is an important physiological modulator. The pH theory", in *Carbonic Anhydrase and Modulation of Physiologic and Pathologic Processes in the Organism*, I. Puscas, Ed., Helicon, Timisoara **1994**, pp. 147-179.

**Received: June 2, 1997 - Accepted: June 12, 1997 - Received in revised camera-ready format: June 13, 1997**

